# Brain SIRT1 Mediates Metabolic Homeostasis and Neuroprotection

**DOI:** 10.3389/fendo.2018.00702

**Published:** 2018-11-23

**Authors:** Jing Xu, Charlie W. Jackson, Nathalie Khoury, Iris Escobar, Miguel A. Perez-Pinzon

**Affiliations:** ^1^Cerebral Vascular Disease Research Laboratories, Department of Neurology, Leonard M. Miller School of Medicine, University of Miami, Miami, FL, United States; ^2^Department of Neurology, Leonard M. Miller School of Medicine, University of Miami, Miami, FL, United States

**Keywords:** Sirt1, obesity, type 2 diabetes mellitus, circadian rhythms, cerebral ischemia, Alzheimer's disease, Parkinson's disease

## Abstract

Sirtuins are evolutionarily conserved proteins that use nicotinamide adenine dinucleotide (NAD^+^) as a co-substrate in their enzymatic reactions. There are seven proteins (SIRT1-7) in the human sirtuin family, among which SIRT1 is the most conserved and characterized. SIRT1 in the brain, in particular, within the hypothalamus, plays crucial roles in regulating systemic energy homeostasis and circadian rhythm. Apart from this, SIRT1 has also been found to mediate beneficial effects in neurological diseases. In this review, we will first summarize how SIRT1 in the brain relates to obesity, type 2 diabetes, and circadian synchronization, and then we discuss the neuroprotective roles of brain SIRT1 in the context of cerebral ischemia and neurodegenerative disorders.

## Introduction

Sirtuins are homologs of yeast silent information regulator 2 (Sir2). Sir2 has attracted the attention of researchers given its involvement in longevity (1). The mammalian sirtuins (SIRT1-7) have different subcellular localizations. SIRT1, SIRT6, and SIRT7 are mainly localized in the nucleus, whereas SIRT1 is also reported to translocate in the cytoplasm. SIRT2 is predominantly cytoplasmic and shuttles to the nucleus transiently. The mitochondrial sirtuins are SIRT3, SIRT4, and SIRT5 (2). In terms of enzymatic activities, sirtuins share a conserved nicotinamide adenine dinucleotide (NAD^+^) binding site and remove acetyl groups from target proteins in an NAD^+^-dependent manner. Additionally, some sirtuins have been reported to exhibit demyristoylase (SIRT2), ADP-ribosyltransferase (SIRT4 and SIRT6), and demanlonylase and desuccinylase (SIRT5) activities (2).

### The role of SIRT1 as a metabolic sensor

Among all sirtuins, SIRT1 is the most extensively studied and well-characterized. As mentioned above, SIRT1 is an NAD^+^ dependent deacetylase that removes the acetyl groups from protein substrates to add to the ADP-ribose, a product from the cleavage of NAD^+^. NAD^+^ is a dinucleotide with one nucleotide contains an adenine and the other contains nicotinamide (3). In addition to be the rate-limiting co-substrate for NAD^+^ dependent enzymes, NAD^+^ can be used a coenzyme in the metabolic redox reactions. NAD^+^ exists in two forms, the oxidized form as NAD^+^, and the reduced form as NADH. NAD^+^/NADH plays a critical role in glycolysis and cellular respiration for ATP production. In glycolysis, NAD^+^ is reduced to NADH. In oxidative phosphorylation and cellular respiration, NADH is oxidized to NAD^+^ by electron transport chain (ETC) (1). As such, NAD^+^ concentrations fluctuate with cellular metabolic status and nutrient availability. NAD^+^ levels increased during the energetic crisis, such as calorie restriction and decreased under conditions of high-energy load, such as high-fat diets. The fact that Sirt1 enzymatic activity depends upon NAD^+^ levels allows Sirt1 to act as a metabolic sensor that couples cellular metabolic status to regulatory responses (1).

### SIRT1 in metabolism

SIRT1 is widely distributed in the body and plays diverse roles in metabolism in different organs including liver, pancreas, muscle, and adipose tissue (4–6). One of the important aspects associated with increased SIRT1 activity is the caloric restriction (CR) (7, 8). CR has been extensively studied, where it has been demonstrated that SIRT1 plays a central role in CR-induced longevity (8–11). As mentioned, it has been suggested that SIRT1, as a metabolic sensor, coordinates the transcriptional networks with the restricted metabolic status (8, 12, 13). During times of energy reduction, NAD^+^ concentrations increase, thereby enhancing the NAD^+^ deacetylase activity of SIRT1. The SIRT1 mediates deacetylation of a broad range of protein substrates. Proteins that regulate mitochondrial biogenesis, glucose homeostasis, inflammation, and apoptosis have been identified as SIRT1 substrates (14–16). These biological functions are linked to energy homeostasis and eventually extend lifespan.

### SIRT1 in the brain

**Figure 1:**
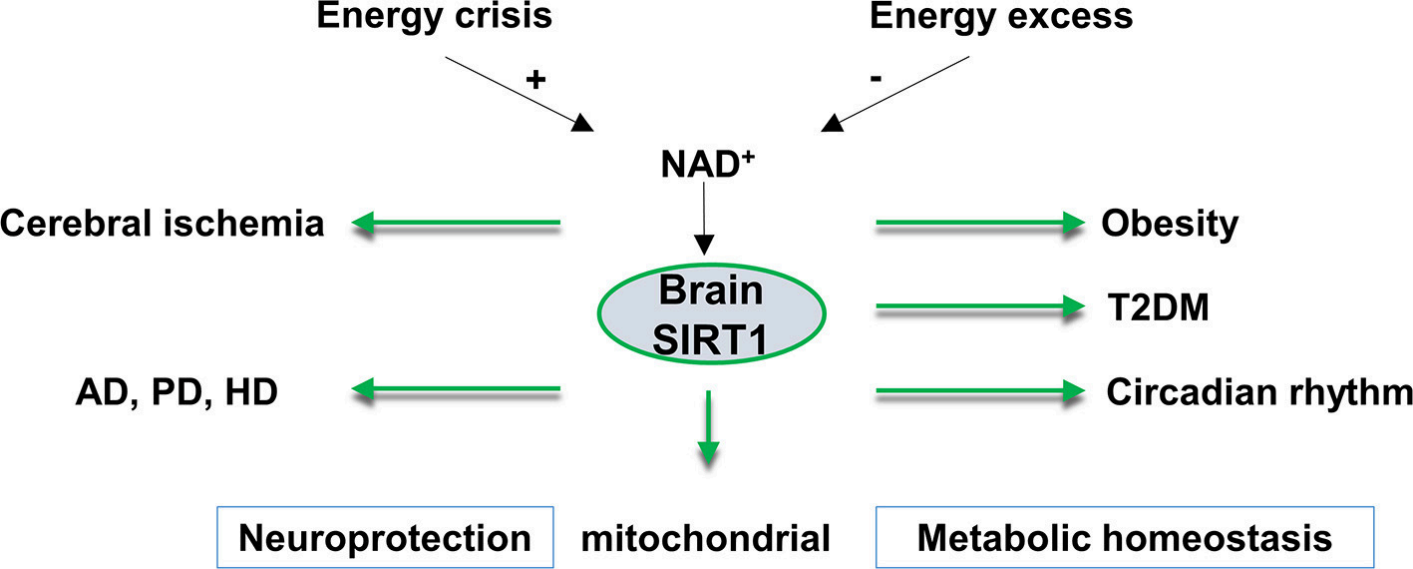
An overview of brain SIRT1 in metabolic and neurological disorders. Brain SIRT1 activity is dependent on NAD^+^ levels, which increases under energy crisis, and decline with high energy load. Any dysregulation of brain SIRT1 activity can have devastating consequences in terms of mitochondrial function, metabolic homeostasis, circadian synchronization, and neurological function. A proper function of brain SIRT1 is protective against obesity, diabetes, circadian dysregulation. In addition, brain SIRT1 exerts neuroprotection against ischemic injury and neurodegenerative disorders, such as Alzheimer's disease (AD), Parkinson's disease (PD), and Huntington's disease (HD). T2DM, type 2 diabetes, NAD^+^, nicotinamide adenine dinucleotide.

SIRT1 is widely expressed in the adult brain. Most of the SIRT1 is localized in the neuronal nuclei. However, SIRT1 is also found in the glial cells of post-mortem human brains, and in neural stem cells, microglia, and astrocytes in culture (17). In the hypothalamus, the control center for homeostasis, SIRT1 mRNA is highly expressed in the arcuate, ventromedial, dorsomedial and paraventricular nuclei of the hypothalamus, which suggests an important role for brain SIRT1 in regulating metabolic status (18). Another function of brain SIRT1 is the regulation of the central circadian clock in the suprachiasmatic nucleus (SCN) of the hypothalamus. In the SCN of the hypothalamus, SIRT1 regulates circadian clock gene expressions by mediating the acetylation status of circadian genes (19). Besides the physiological functions of SIRT1 in the hypothalamus, SIRT1 is reported to exert neuroprotection in neurological dysfunctions (20). In this review, we will focus on the roles of SIRT1 in the brain in metabolism, circadian rhythm, and SIRT1 function in the context of cerebral ischemia and neurodegenerative disorders (Figure 1).

## SIRT1 and mitochondrial functions

Mitochondrial is one of the most important sources for cellular energy in eukaryotes, producing up to 95% of the ATP through oxidative phosphorylation (21). This provides great significance into the roles of the mitochondrial in the brain, where it is estimated to take up to 20% of the total oxygen consumption of the body energy (22). As such, this dysfunction could affect metabolic efficiency, thus linking to a common pathology ranging from metabolic disorders to neurological diseases (21).

SIRT1 can regulate the transcription of mitochondrial genes encoded in the nucleus that are involved in vital mitochondrial processes related to longevity and aging. While SIRT1 is mainly localized in the nucleus, levels have also been detected in the mitochondrion where it may interact with different substrates (23). In this section, we will discuss SIRT1's involvement in important mitochondrial functions including mitochondrial biogenesis, mitophagy, and energy metabolism.

### Regulation of insulin secretion by SIRT1 via UCP2

Uncoupling protein 2 (UCP2) is an inner mitochondrial membrane protein that can uncouple oxidative phosphorylation from respiration/ATP production. This is done via dissipation of the proton gradient, in which protons are returned to the mitochondrial matrix. UCP2 is found in many different tissues, including the brain, and has been shown to be involved in energy balance (24), homeostasis, and longevity (25). SIRT1 has been found to positively regulate insulin production by means of repressing UCP2 (26–28). As a result, cells express higher ATP levels after glucose stimulation, which is essential for inducing insulin secretion (26). In this manner, levels of insulin are regulated commensurate to levels of food intake and if impaired may contribute to obesity-induced diabetes (26). In this respect, SIRT1 can respond to nutrients available in the environment and promote transcriptional changes that may enhance energy metabolism.

### PGC-1α and SIRT1 interact to induce mitochondrial biogenesis and metabolic processes

Peroxisome proliferator-activated receptor γ (PPARγ) coactivator-1α (PGC-1α) is a transcriptional coactivator and major regulator of mitochondrial biogenesis and several metabolic processes (29). Studies have shown that SIRT1 interacts with PGC-1α to induce its transcriptional activity via deacetylation (16, 30–32). PGC-1α may activate a wide array of transcription factors (TFs) that include both DNA-binding TFs, such as nuclear respiratory factor 1 (NRF-1), and nuclear hormone receptors, such as PPARγ, thyroid hormone receptors, retinoic acid receptors, glucocorticoid receptors, and estrogen receptors (33, 34).

NRF-1, specifically, can regulate the activation of the nuclear-encoded mitochondrial transcription factor A (TFAM), which can bind to mtDNA and stimulate mitochondrial DNA replication and increase the expression of mitochondrial genes (35). As a result, NRF-1 induces the expression of mitochondrial transporters, components of oxidative phosphorylation, and ribosomal proteins (36). Aquilano et al. found that SIRT1 and PGC-1α also interact with TFAM within the mitochondria (23). In either case, the subsequent increased expression of mitochondrial genes promotes mitochondrial biogenesis—an essential process important for maintaining oxidative capacity and levels of energy production. In some instances, SIRT1 activity is required to stimulate mitochondrial biogenesis, as reported in pulmonary arteriolar smooth muscle cells (37); however, whether SIRT1 is necessary for mitochondrial biogenesis to occur has recently become controversial (38).

The increase of mitochondrial gene expression also stimulates several metabolic processes depending on the tissue type (33, 36). In the brain and heart, PGC-1α functions as an important regulator of the metabolism of reactive oxygen species (ROS) under normal physiological conditions and certain states of oxidative stress (36). In conditional liver-specific SIRT1 KO mice, PPARα signaling activated by PGC-1α was impaired (39). This lead to a decrease in fatty acid oxidation and ketogenesis, suggesting a vital role for SIRT1 in regulating hepatic lipid homeostasis (39). Dysregulation of any of these processes may contribute to both aging and age-associated metabolic diseases.

### SIRT1 and mitophagy

Maintaining quality mitochondrial pools is essential for cell health and viability. Mitochondrial components are typically damaged by the accumulation of ROS—a byproduct of the mitochondrial electron transport chain—which typically occurs during conditions of stress. Due to its close proximity, ROS overproduction damages mtDNA that may ultimately contribute to neurodegenerative disorders, stroke, cancer, and age-related diseases (40–42). Constant mitochondrial turnover is important to maintain a healthy mitochondrial population. Thus, a quality control mechanism is required to eliminate and replace dysfunctional mitochondria with new and more efficient mitochondria. To achieve this specialized form of homeostasis in response to stress, cells utilize a process known as mitophagy for the selective degradation of mitochondrial components. This elimination process is balanced with mitochondrial biogenesis. While the exact mechanism for mitophagy has yet to be elucidated, some studies indicate PINK1/PARKIN as the key pathway involved (43, 44). As discussed above, SIRT1 is essential for promoting mitochondrial biogenesis; however, it also plays an important role in autophagy.

**Figure 2:**
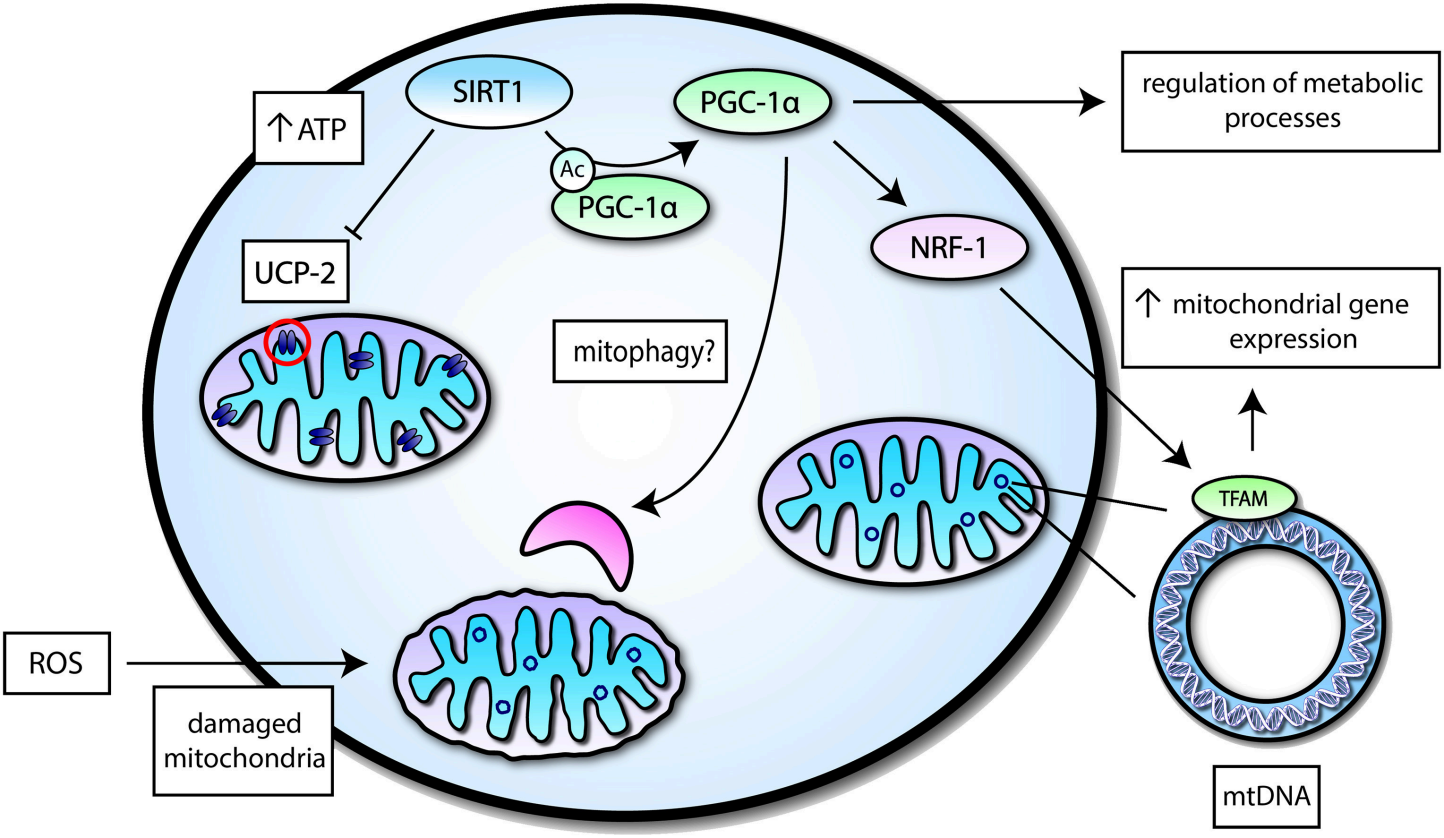
A simplified overview of mitochondrial functions mediated by SIRT1 activity. SIRT1 may interact with transcription factors or mitochondrial proteins to induce different effects related to mitochondrial function—a select few of these proteins are highlighted. SIRT1 can suppress Uncoupling protein 2 (UCP-2) in the inner mitochondrial membrane to increase levels of ATP, which is important for energy metabolism. SIRT1 may also deacetylate Peroxisome proliferator-activated receptor γ (PPARγ) coactivator-1α (PGC-1α) to induce its activation and augment mitochondrial biogenesis by increasing mitochondrial gene expression via Nuclear respiratory factor 1 (NRF-1) and Nuclear-encoded mitochondrial transcription factor A (TFAM). PGC-1α itself can regulate different metabolic processes and may potentially play a role in mitophagy.

Several studies have shown that SIRT1 interacts with components of the autophagy machinery including Forkhead box O3 (FOXO3)—a transcription factor heavily associated with autophagy induction (45–47). In the aged kidney, the mitochondrial damage was associated with deficiencies in SIRT1, and under hypoxic conditions, SIRT1 was able to promote cell adaptation by deacetylation of FOXO3 (46). Another study demonstrated that SIRT1 is essential for fully activating autophagy under conditions of starvation (48). Additionally, SIRT1 deficient embryos and neonatal mice displayed an accumulation of abnormal organelles, especially mitochondria, and impaired autophagy (48). PGC-1α is also involved in mitophagy as it regulates the expression of transcription factor EB (TFEB)—a well-known master regulator of autophagy and lysosomal biogenesis (49). It may be possible that SIRT1 may also influence mitophagy given its interaction with PGC-1α. Taken together, SIRT1 is involved in diverse mechanisms for the regulation of mitochondrial functions (Figure 2). The regulation of SIRT1 in mitochondrial functions may underlie its importance in regulating energy metabolism and in so doing, may be part of its neuroprotective role.

## Brain SIRT1 and obesity

The hypothalamus is the control center for homeostasis. In the arcuate nucleus of the hypothalamus, the proopiomelanocortin (POMC) neurons suppress appetite while the activation of agouti-related peptide/neuropeptide Y (AgRP/NPY) neurons stimulate appetite (50). The ventromedial nucleus (VMN) is another nucleus involved in satiety as VMN lesions lead to an increase in food intake and obesity (51). In recent years, a substantial number of studies demonstrated that the hypothalamic SIRT1 is crucially important for the central regulation of food intake and energy expenditure.

SIRT1 in the POMC neurons is required to protect against high calorie-induced obesity. When challenged with a hypercaloric diet, POMC-SIRT1 mutant mice showed reduced energy expenditure and increased body weight (52). Interestingly, these metabolic changes were not due to hypoactivity, as the mutant mice showed unaltered levels of daily activities compared to their control counterparts (52). The reduction in energy expenditure could be explained by a reduction in sympathetic nerve activity in the adipose tissue of the mutant mice (52). Conversely, mice with overexpressed SIRT1 in the POMC neurons exhibited a leaner phenotype compared to their wild-type littermates (53). Age-related weight gain was absent in the POMC-SIRT1 overexpressed mice. The leaner phenotype was attributed to increased sympathetic activity in the adipose tissue with consequently enhanced energy expenditure (53).

Another mechanism in which SIRT1 modulates systemic homeostasis is through deacetylation of Forkhead box protein O1 (FoxO1). FoxO1 is a downstream transcription factor in the insulin signaling pathway. Hypothalamic FoxO1 activation or overexpression inhibits the anorexigenic effects of insulin (54), increases adiposity, and leads to weight gain (55). The overexpression of SIRT1 in POMC neurons was able to rescue FoxO1 activation induced obesity (55). These effects occurred through decreased acetylation and expression of FoxO1 by POMC-SIRT1 overexpression (55). Similarly, FoxO1 mediated hyperphagia was blunted by hypothalamic SIRT1 overexpression (56).

Hypothalamic SIRT1 is also implicated in the leptin-mediated regulation of metabolism. Leptin is a hormone secreted by the adipose tissue that suppresses body weight. In the hypothalamus, leptin binds to its receptor (Ob-Rb) and activates the signal transducer and activator of transcription 3 (STAT3), which further regulates gene expressions to affect energy homeostasis. The leptin-induced protective mechanisms against obesity are dependent on SIRT1 in the POMC neurons. In POMC-SIRT1 deficient mice, leptin-mediated activation of the phosphatidylinositol-4,5-bisphosphate 3-kinase (PI3K) signaling pathway and the suppression of food intake were disrupted (52). In addition, when SIRT1 is overexpressed in the hypothalamus, either in POMC or AgRP neurons, non-obese mice exhibited increased sensitivity to leptin, as demonstrated by increased phosphorylation of STAT3 as well as reduced food intake (53). Interestingly, these phenotypes were blunted in mice consuming a high-fat diet, due to decreased expression of SIRT1 and NAD^+^ levels in the hypothalamus, suggesting that the metabolic status could influence the function of hypothalamic SIRT1 (53). In support of this, the hypothalamic SIRT1 expression is induced upon feeding in the standard fed mice, whereas diet-induced obesity abrogated this induction (56). In addition to the arcuate nucleus, SIRT1 in steroidogenic factor 1 (SF1) neurons of the VMN also contributes to the physiological function of leptin. The lack of SIRT1 in the SF1 neurons predisposed mice to dietary-induced obesity. SF1-SIRT1 mutant mice exhibited diminished energy expenditure and impaired leptin sensitivity (57). In contrast, SIRT1 overexpression in the SF1 neurons restored oxygen consumption, increased leptin sensitivity and protected mice against high-calorie diet induced weight gain (57).

**Figure 3:**
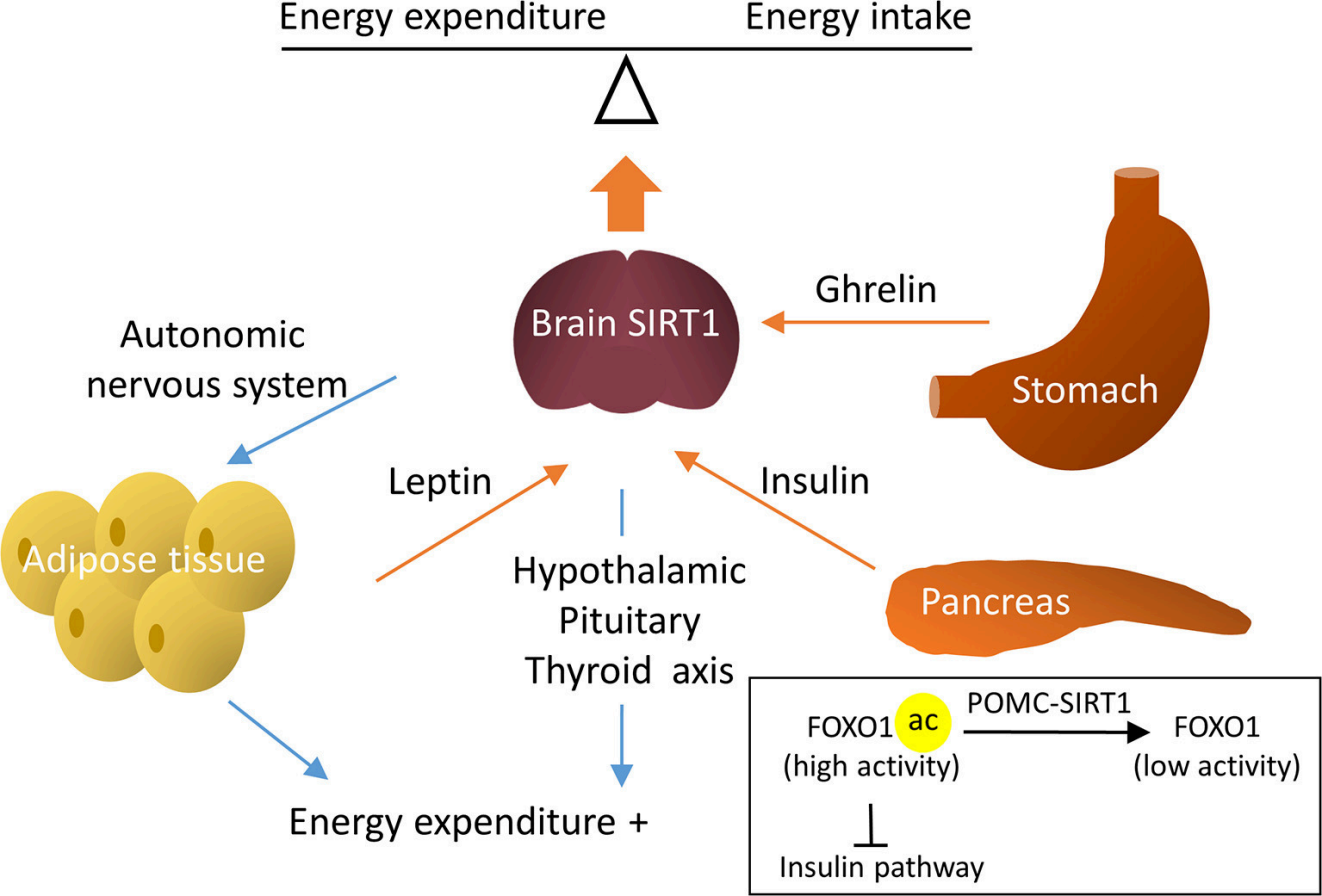
Regulative mechanisms of brain SIRT1 in metabolic homeostasis. Brain SIRT1 increases energy expenditure via the hypothalamic pituitary thyroid axis and increased sympathetic nerve activity in the adipose tissue. In addition, hormones, such as leptin, insulin, and ghrelin through brain SIRT1 to balance energy expenditure and energy intake. For example, SIRT1 in POMC neurons deacetylate Forkhead box protein O1 (FOXO1) to increase the insulin signal pathway.

In the aforementioned studies, the activation of hypothalamic SIRT1 negatively regulated energy balance and protected against obesity. Contrary to these findings, studies also reported a positive energy regulation by brain SIRT1. Apart from leptin, ghrelin is another peptide released peripherally and acts on the central nervous system (CNS) to regulate metabolism. Ghrelin is produced by the stomach and activates AMPK in the hypothalamus to increase appetite. In rodents, the intracerebroventricular (ICV) infusion of the SIRT1 inhibitor, EX-527, blunted the ghrelin-induced food intake, thus demonstrating that ghrelin is dependent on hypothalamic SIRT1 to stimulate appetite (58, 59). Similar beneficial metabolic effects were seen in two other studies that blocked brain SIRT1 by the ICV infusion of EX-527. One study showed that the inhibition of brain SIRT1 in fasted rats could reduce food intake and decrease weight gain (60). The other study compared the effects of brain SIRT1 inhibition in obese and lean rats. The authors observed a significant decrease in body weight and an increase in energy expenditure in diet-induced obese rats, but not in rats fed normal chow upon brain SIRT1 inhibition (61). The mechanisms were attributed to increased activity of the hypothalamic-pituitary-thyroid axis, resulting in enhanced energy expenditure (61). The discrepancy between the hypophagia and hyperphagia effects by SIRT1 activation may be due to the different animal models used in these studies (i.e., ICV infusion of EX-527 and genetic deletion of SIRT1 in a specific population of neurons). Despite the controversies, these studies demonstrated a crucial role for brain SIRT1 in the systematic regulation of energy homeostasis (Figure 3).

## Brain SIRT1 and type 2 diabetes

Obesity is a leading risk factor for type 2 diabetes (T2DM). The accumulation of fat, especially visceral fat, progressively enhances insulin resistance and eventually leads to T2DM (62, 63). Given the crucial role of SIRT1 in obesity, it is no surprise that emerging evidence suggests SIRT1 within the brain controls the systematic regulation of glucose/insulin homeostasis. Mentioned briefly above, SIRT1 in SF1 neurons is required for the defense against dietary-induced obesity. In addition to this, insulin activated PI3K signaling was blunted in the skeletal muscle of SF1-neuron-SIRT1 deleted mice. Conversely, SIRT1 overexpression in SF1 neurons enhanced skeletal muscle insulin sensitivity in these mice (57).

Resveratrol is a potent SIRT1 activator that improves glucose homeostasis. Studies demonstrate that brain SIRT1 at least partially contributes to the resveratrol-mediated glucose balance (64). ICV infusion of resveratrol rescued the hyperglycemia phenotype in diet-induced obese and diabetic mice (64). In support of the former data, hypothalamic or systemic administration of resveratrol increased hepatic insulin sensitivity, which was blunted by the inhibition of SIRT1 in the hypothalamus (65). Collectively, these data suggest SIRT1 activation within the brain is likely to improve insulin resistance and combat against diabetes. In such cases, brain SIRT1 activation leads to suppressed peripheral glucose production.

Two studies that investigated the cell type-specific role of neuronal SIRT1 in glucose metabolism suggested a different regulatory mechanism. Neuronal SIRT1-deficient mice exhibited higher insulin sensitivity in the hypothalamus and peripheral tissue. It was suggested that SIRT1 deacetylates and represses Insulin receptor substrate 1 (IRS-1) and the insulin signaling pathway. In this case, central PI3K signaling was enhanced in neuronal SIRT1 deficient mice (66). Another study demonstrated that neuronal SIRT1 mediates glycolysis in the brain. Pharmacological inhibition or genetic mutation of neuronal SIRT1 caused glycolysis deficits *in vitro* and *in vivo*, whereas resveratrol treatment increased the glycolysis rate in primary neurons (67). It is reported that, in peripheral tissues, SIRT1 inhibits glycolysis to reduce glucose consumption (68). For example, in liver, under metabolic stress, SIRT1 deacetylates and activates PGC-1α to suppresses glycolysis and promote gluconeogenesis. These data demonstrate that SIRT1 regulates glucose metabolism in a tissue-specific and cell type-specific manner (16).

## Brain SIRT1 and circadian rhythm

The circadian rhythm is a 24 h endogenous cycle that allows organisms to synchronize their physiology and behavior to the daily cycle of daylight and darkness (69, 70). The circadian clock is entrainable by internal and external zeitgebers “time givers.” In mammals, the circadian clock is found across different tissues, yet the central clock is found in the SCN of the hypothalamus from which it entrains peripheral clocks to regulate oscillatory functions, such as metabolism and the sleep/wakefulness cycle (69, 70).

The molecular mechanism of the circadian rhythm consists of a set of transcriptional activators and repressors involved in positive and negative autoregulatory feedback loops (69, 71). In mammals, the core clock genes are the acetyltransferase CLOCK (Circadian Locomotor Output Cycles Kaput) and its heterodimer BMAL1 (Brain and muscle Arnt-like protein-1). When dimerized, the CLOCK-BMAL1 complex translocates to the nucleus and induces the expression of several downstream genes. Among these genes are their own negative regulators period (PER1, PER2, PER3) and cryptochrome (CRY1 and CRY2) proteins (69, 70). Over the course of the day, PER and CRY start to accumulate and together with the casein kinase 1δ (CK1δ) and CK1ε translocate to the nucleus to repress their own transcription (70). As repression progresses, PER and CRY levels decline and transcription by CLOCK-BMAL1 re-initiates a new cycle (70). PER and CRY are also eliminated by post-translational modifications and degradation (69, 70). The CLOCK-BMAL1 complex also regulates the downstream retinoic acid-related orphan receptors (RORα, RORβ) and the nuclear receptors (Rev-Erbα, Rev-Erbβ), which compete for the regulation of the BMAL1 promoter and reinforce the oscillation (70).

### SIRT1 in the peripheral clocks

**Figure 4:**
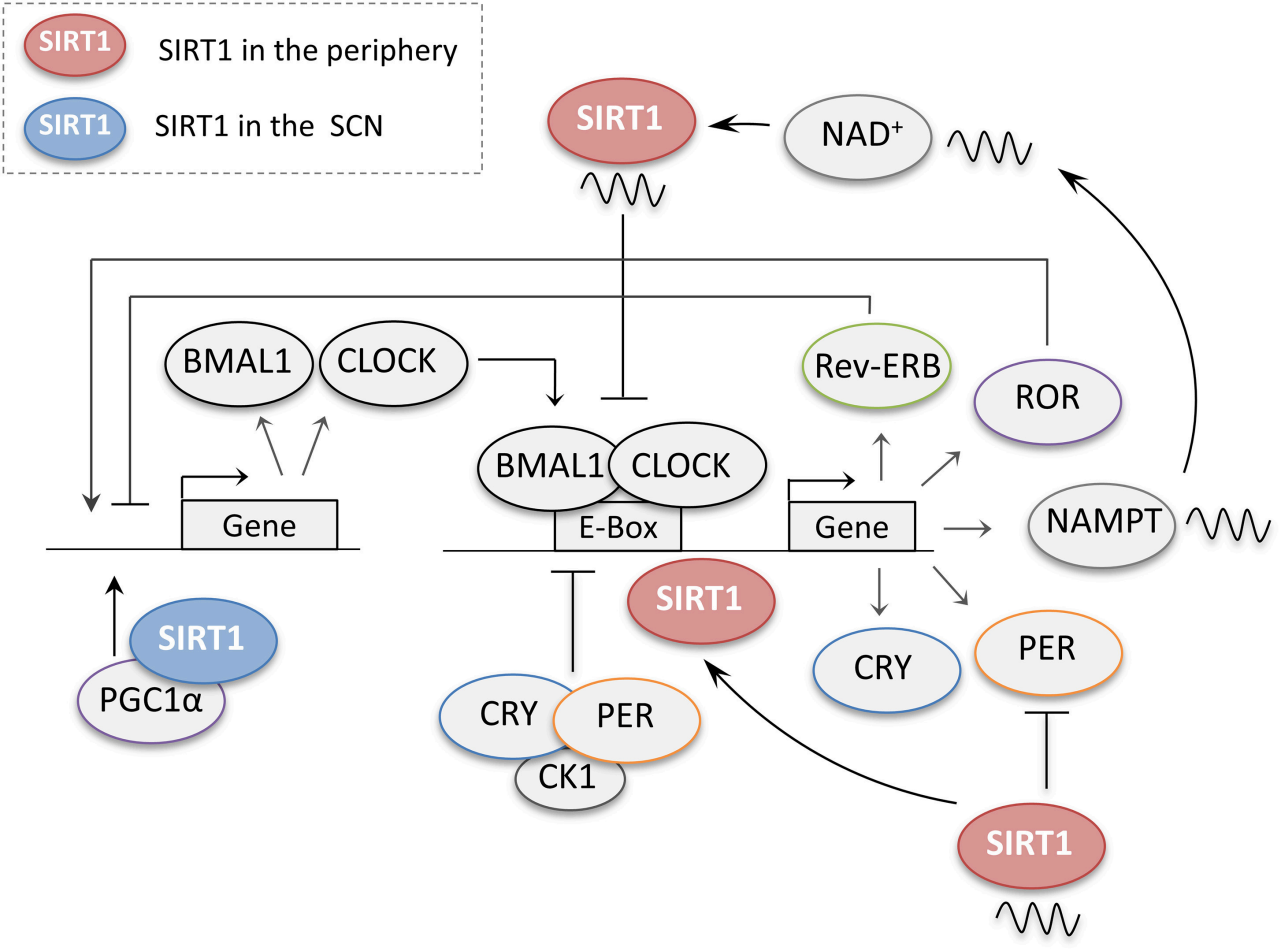
The regulation of central and peripheral clock genes by SIRT1. When dimerized, the core clock genes CLOCK and BMAL1 promote the expression of several downstream genes including their own negative regulators periods (PER) and cryptochromes (CRY). PER and CRY accumulate during the day and together with casein kinase 1 (CK1) then repress their own transcription. The CLOCK-BMAL1 complex also regulates the retinoic acid-related orphan receptors (ROR) and the nuclear receptors (Rev-Erb), which compete for the regulation of the BMAL1 promoter. In the peripheral clocks, SIRT1 regulates the circadian genes at different levels. SIRT1 protein levels cycle in a circadian manner, and through its rhythmic binding to the CLOCK-BMAL1 complex SIRT1 promotes the circadian transcription of Bmal1, Rorγ, Per2, and Cry1. SIRT1 also promotes the deacetylation and degradation of the PER2 protein. SIRT1 activity has also been reported to cycle in a circadian manner owing to the rhythmic expression of NAMPT, a crucial enzyme for NAD^+^ biosynthesis, by the CLOCK-BMAL1 complex. In turn, SIRT1 also acts as a negative regulator of the CLOCK-BMAL1 complex thus preventing the activation of circadian promoters. In the suprachiasmatic nucleus (SCN), SIRT1 activates the transcription of the circadian genes BMAL1 and CLOCK through PGC-1α.

SIRT1 has been shown to be an important regulator of the circadian clock genes in both the central and peripheral clocks (Figure 4). In 2008, two independent studies using peripheral tissues were the first to link SIRT1 to the regulation of the clock genes. Using mouse hepatocytes and cultured fibroblasts, it was shown that the protein levels of SIRT1 cycle in a circadian manner, in turn, is required to promote the circadian transcription of Bmal1, Rorγ, Per2, and Cry1 (19). This study also showed that the binding of SIRT1 to the CLOCK-BMAL1 complex is rhythmic and promotes the deacetylation and degradation of the PER2 protein (19). In the second study using fibroblasts and liver tissues, SIRT1 was reported to be a negative regulator of the CLOCK-BMAL1 complex (72). By antagonizing the acetyltransferase activity of CLOCK, SIRT1 removes acetyl-marks from histone H3 and BMAL1, preventing the CLOCK-BMAL1 heterodimer from activating circadian promoters. This study also revealed that SIRT1's activity rather than levels is regulated in a circadian manner (72). Subsequent studies then revealed that the rhythmic activity of SIRT1 is due to the oscillatory patterns of NAD^+^ levels regulated by its rate-limiting enzyme NAMPT, which is positively regulated by the core clock genes CLOCK-BMAL1 (73, 74). The activation of SIRT1 through this NAMPT-mediated NAD^+^ biosynthetic pathway, in turn, inhibits the activity of CLOCK-BMAL1, thus forming a negative feedback loop (73, 74). These studies revealed a crucial role for SIRT1 in coupling metabolism to the circadian cycle through its reliance on NAD^+^ as a cofactor. Supporting this observation, studies have shown that high-fat diets can disrupt the rhythmicity of circadian clock genes in several tissues and that the administration of SIRT1 activators such resveratrol, can reverse these effects and restore the rhythmicity to the circadian genes (75, 76). Additionally, several other studies have attributed different functions to SIRT1 in the regulation of the circadian clock genes in peripheral tissues thus adding additional levels of complexity to its function (77–79).

### SIRT1 in the central clock

In the SCN of the hypothalamus, SIRT1 was reported to activate the transcription of the circadian genes BMAL1 and CLOCK through PGC-1α (80) (Figure 4). Interestingly, this study also showed that aging reduces the levels of SIRT1 in the SCN, which coincided with reduced BMAL1 and PER2 levels. This, in turn, leads to a longer intrinsic period and disruption in the activity patterns and entrainment of mice to the light schedule. Furthermore, the knockout of *Sirt1* from young mice brains was able to phenocopy these age-dependent disruptions in the circadian cycle, while its overexpression protected old mice from the age-dependent effects (80). Thus, this study revealed a crucial role for SIRT1 in the activation of the central pacemaker and maintenance of robust circadian control in young animals. It also suggested that the age-dependent reduction in SIRT1 led to the observed disruptions in the circadian cycle with aging (80). Another interesting study showed that SIRT1 from the ventromedial hypothalamus (VMH) sends nutrient-time information to the central clock through efferent signals to synchronize the central clock to feeding cues (81). SIRT1 ablation from the SF1 neurons of the VMH disrupted the connection between food intake and circadian rhythm as revealed by deregulated activity behaviors and circadian gene expression in the SCN (81). This study strongly supports the role of SIRT1 as a nutrient sensor that couples metabolism to the circadian rhythm of the central clock.

**Figure 5:**
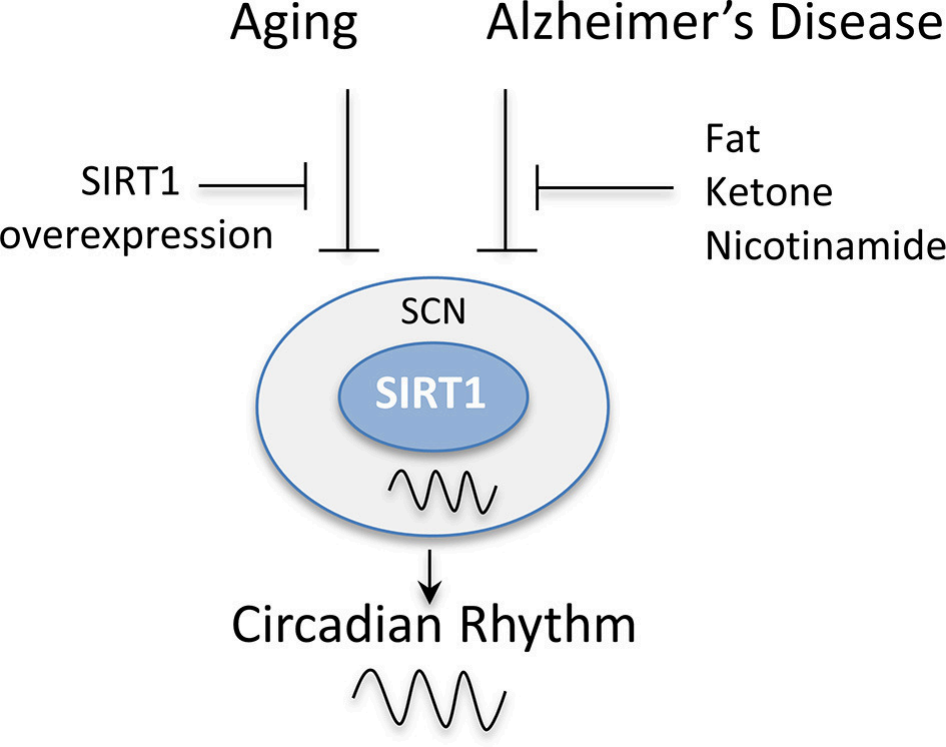
SIRT1 levels in the suprachiasmatic nucleus (SCN) are disrupted by aging and Alzheimer's disease. The rhythmic expression of SIRT1 in the SCN has been reported to be disrupted in animal models of aging and Alzheimer's disease. This, in turn, disrupts the circadian expression of clock genes causing a disruption in the activity patterns of mice and their entrainment to light. The overexpression of SIRT1 protected mice from these age-dependent effects. Similarly, the administration of fat, ketone bodies, or nicotinamide rescued the circadian expression of clock genes in Alzheimer's disease mouse models and restored their locomotor rhythmicity.

The regulation of the circadian genes by SIRT1 in the central clock has also been reported to be disrupted in a number of neurological diseases (Figure 5). It was shown that Apolipoprotein E knockout (ApoE^−/−^) mice, a model of Alzheimer's disease, exhibit disruptions in the circadian locomotor activity under dim light and constant darkness along with impairments in re-entrainment to phase change schedules (82). These mice also exhibit an alteration in the expression of SIRT1 and circadian clock genes in the SCN (82). Interestingly, the supplementation with fat or ketone bodies or the intraperitoneal administration of nicotinamide can rescue the circadian clock in these mice by restoring their locomotor rhythmicity and circadian expression of SIRT1 and clock genes (82). Additionally, in triple transgenic Alzheimer's disease (3 × Tg-AD) mice, the patterns of expression of circadian clock genes were also reported to be disrupted in the SCN in response to daylight and darkness. Consistently, these mice also exhibited significantly higher levels of SIRT1 in the SCN compared to non-transgenic after a 12 h exposure to darkness (83). Thus, these studies combined suggest that SIRT1 may be a relevant therapeutic target for the restoration of the circadian rhythm in the SCN, which is disrupted in both aging and neurological disorders.

## Sirt1 and cerebral ischemia

Researchers have established different roles for brain SIRT1 in different neurological diseases. Evidence from preclinical studies established a neuroprotective role for SIRT1 in ischemic injury. SIRT1 deficient mice, compared to their wild-type littermates, exhibited significantly larger infarct volume and increased impairment of neurological functions after permanent middle cerebral artery occlusion (pMCAO) (84). In a similar line of evidence, pharmacological blockade of SIRT1 activity by SIRT1 inhibitor sirtinol increased the infarct volume following pMCAO (84). In contrast, SIRT1-overexpression protected the brain from cerebral ischemic injury. In a bilateral common carotid artery stenosis (BCAS) model that causes chronic cerebral hypoperfusion, wild-type mice displayed white matter deficits and spatial memory impairments following BCAS (85). Conversely, SIRT1 overexpressed mice showed preserved histological outcome of the corpus callosum and restored spatial working memory (85). Additionally, increased SIRT1 activity by Activator 3, a specific SIRT1 activator, reduced infarct volume in mice (84).

The neuroprotection against cerebral ischemia by SIRT1 is achieved through multiple mechanisms. Following ischemia, stressors, such as DNA damage and oxidative stress activate the tumor suppressor gene, p53, which mediates apoptosis (86). Ischemia-induced activation of p53 triggers the mitochondrial apoptotic pathway and facilitates neuronal cell death (86, 87). Inhibition of p53 blocks apoptosis, promotes a survival signaling pathway, and protects neurons against ischemic-induced cell death (88, 89). Genetic deletion or pharmacological inhibition of SIRT1 increased the acetylation of p53 in the peri-infarct area (84). In contrast, SIRT1 activation deacetylated p53 and reduced p53-dependent neuronal apoptosis (90).

SIRT1 dependent endothelial nitric oxide synthase (eNOS) modulation is another beneficial mechanism. Nitric oxide (NO) is a vasodilatory factor that is produced by endothelial nitric oxide synthase (eNOS) in endothelial cells. Acetylated eNOS was significantly increased at 2 h after BCAS in wild-type mice, whereas in SIRT1-Tg mice, the acetylation of eNOS was not observed (85). Increased deacetylation of eNOS is suggested to increase NO production, regulating the vascular tone of blood vessels, and helping to maintain cerebral blood flow during chronic hypoperfusion (85). Consistent with this, in a global cerebral ischemia model of bilateral common carotid artery occlusion (BCAO), SIRT1-Tg mice showed significantly preserved cerebral blood flow during BCAO, which was absent in their wild-type littermates. Similar to the former, pharmacological activation of SIRT1 by resveratrol treatment 1 h after MCAO increased plasma NO and decreased infarction volumes in an eNOS dependent manner (91).

As briefly mentioned above, SIRT1 can also mediate protection by retaining the integrity of white matter (85). White matter lesions are commonly seen in elderly people. One study, which enrolled 1,077 subjects, revealed only 5% were completely free of white matter lesions (92). The prevalence of white matter lesions increased with aging and is associated with cognitive defects. Moreover, the cerebral white matter is highly vulnerable to ischemic injuries (93, 94). Of note, evidence also showed that the degree of white matter lesions relates to infarct volumes and predicts future ischemic incidence after the first stroke attack in patients (95). Supporting the white matter protection by SIRT1 in ischemic stroke, studies in other neurological models demonstrate a similar SIRT1 mediated benefit. In neonatal brain injury, SIRT1 regulates glial progenitor cells to promote white matter regeneration (96). Similarly, SIRT1 mediates neuronal protection in an autoimmune model of white matter injury (97).

**Figure 6:**
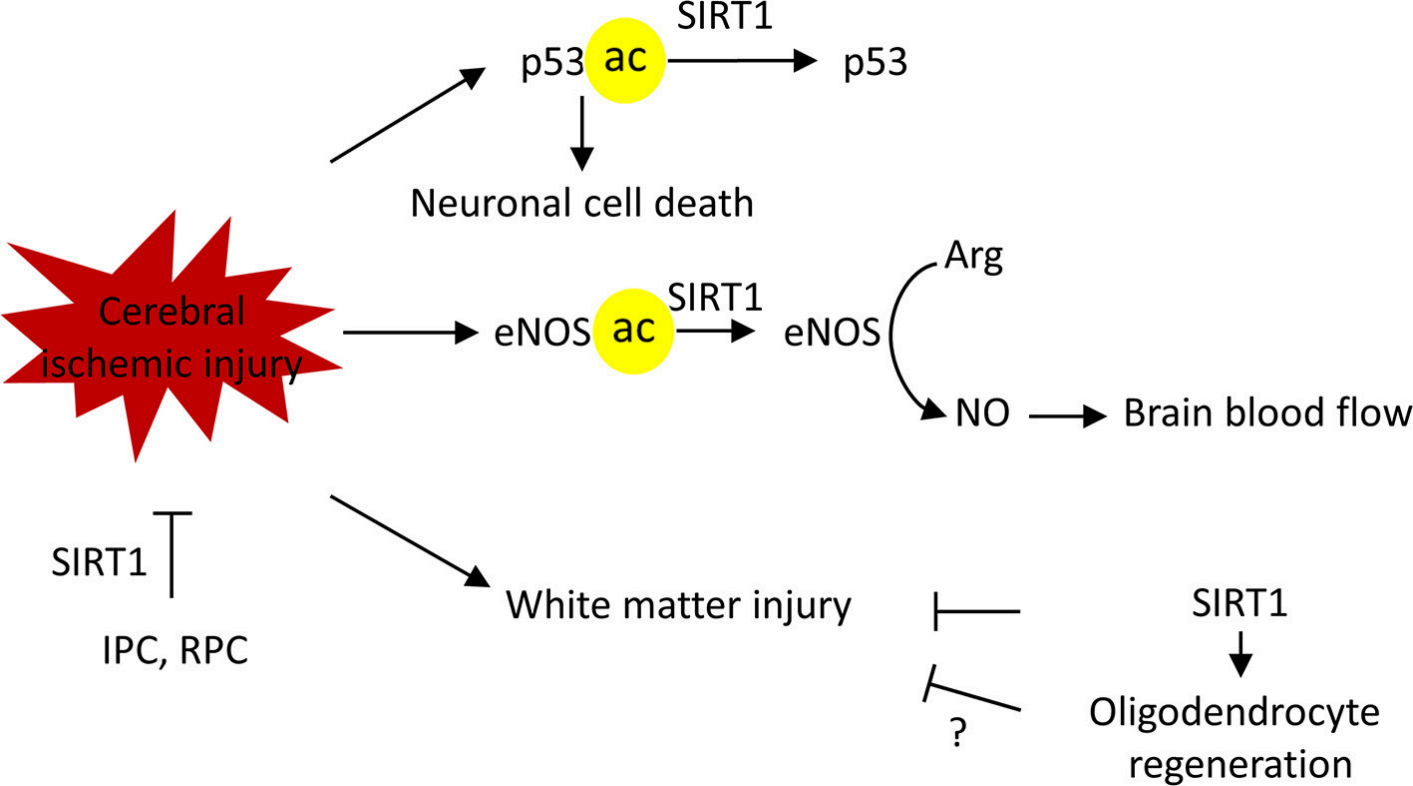
SIRT1 protects against cerebral ischemic injury in multiple mechanisms. SIRT1 deacetylate p53 to block the p53-induced apoptotic pathway, thus, promoting neuronal survival. SIRT1 deacetylates endothelial nitric oxide synthase (eNOS) to regulate vascular tone and maintain brain blood flow. SIRT1protects against white matter injury in ischemic injury, possible via promoting the oligodendrocyte regeneration. Finally, SIRT1 is required for ischemic preconditioning (IPC) and resveratrol preconditioning (RPC) induced ischemic neuroprotection. Arg, L-arginine, NO, nitric oxide.

Importantly, Sirt1 is required in the neuroprotection elicited by ischemic preconditioning (IPC) or resveratrol preconditioning (RPC) (1, 17, 67). IPC develops when a brief period of sublethal ischemia is followed by a period of recovery. It exerts a neuroprotective state against lethal ischemia in different organs of the body including the brain. Furthermore, IPC has shown promising prophylactic potential in diminishing cerebral ischemic injury as shown in recent translational research studies (98, 99). Similar to IPC, resveratrol treatment is able to protect the brain from a following cerebral ischemic attack (1, 17, 67). Collectively, the evidence gathered here demonstrates the pivotal role SIRT1 plays against cerebral ischemia (Figure 6).

## SIRT1 and neurodegenerative disorders

In addition to providing neuroprotection against cerebral ischemia, the activation of SIRT1 has been shown to confer protection against neurodegenerative diseases, such as Alzheimer's disease (AD), Parkinson's disease (PD) and Huntington's disease (HD) (20, 100). These diseases are substantial burdens to society and can be debilitating to afflicted individuals, making it imperative to investigate potential therapeutic factors, like SIRT1.

The benefits of SIRT1 in neurodegenerative diseases was first reported by Graff et al. in a CR model (101). Graff et al. studied an inducible neurodegenerative mouse model, called CK-p25. These mice exhibited a substantial neuronal loss, deficits in synaptic density and plasticity, as well as learning and memory impairments under the induction of doxycycline. CK-p25 mice underwent 3 months of CR and after the sixth week of CR the neurodegeneration was induced. The CR group showed preserved synaptic density, synaptic plasticity, and memory capacities. CR neuroprotection was mediated by SIRT1 activation, shown by the deacetylation of p53, in the CR but not control group. Furthermore, the use of a small SIRT1 activator, as well as SIRT1 overexpression, recapitulated the CR neuroprotection (101). This evidence shows SIRT1's neuroprotective capacity against neurodegenerative effects on synaptic function and memory capacities. In this way, SIRT1 activation, or perhaps overexpression, may protect against synaptic dysfunction in common forms of neurodegeneration.

### Alzheimer's disease and the therapeutic potential of SIRT1

AD is a neurodegenerative disease that can be either early-onset or late-onset. Early-onset is associated with a genetic contribution to the disease's etiology, while the late onset etiology is more complicated and likely multifactorial (102). In the more common, late-onset form of the disease, neuritic senile plaques (NSP) and neurofibrillary tangles (NFT) contribute to neuronal toxicity and death. NSPs originate from the buildup of a protein called β-amyloid (Aβ). β-secretase and γ-secretase are enzymes that cleaves the amyloid precursor protein (APP) to produce Aβ, which is then secreted into the extracellular space, eventually forming toxic aggregates. NFT's are tangles of the cytoskeletal protein tau that receive an aberrant post-translational modification. A common pathological modification of tau is phosphorylation, which forms the toxic p-tau (103). The formation of NSP's and NFT's are pivotal steps in AD pathology.

**Figure 7:**
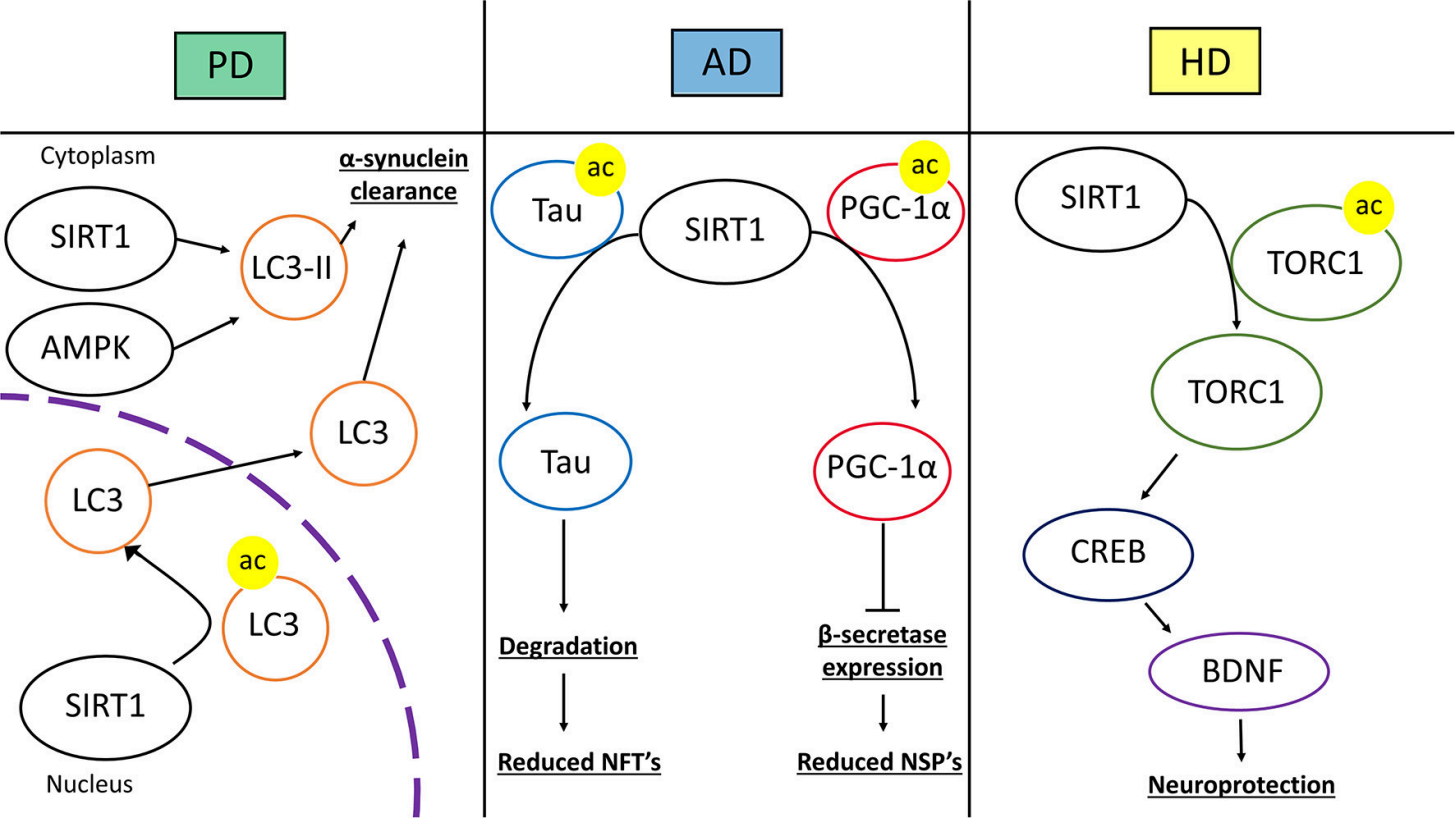
Therapeutic mechanisms of SIRT1 in neurodegenerative disease. The left panel represents SIRT1 in Parkinson's disease (PD). SIRT1 deacetylates microtubule-associated protein 1A/1B-light chain 3 (LC3) in the nucleus which induces the translocation of LC3 into the cytoplasm. In the cytoplasm, SIRT1 and AMP-activated Protein Kinase (AMPK) coordinate to activate LC3-phosphatidylethanolamine (LC3-II). These mechanisms lead to increased autophagic clearance of α-synuclein, reducing α-synuclein deposits. In the middle panel, SIRT1's role in Alzheimer's disease (AD) is represented. SIRT1 can directly deacetylate acetylated-tau protein, increasing its susceptibility to degradation and prevent tau from forming neurofibrillary tangles. SIRT1 can also deacetylate peroxisome proliferator-activated receptor γ (PPARγ) coactivator-1α (PGC-1α), which increases its transcriptional regulation activity. After being deacetylated, PGC-1α can instill transcriptional repression of β-secretase, which in turn can reduce the level of amyloid-β production and neuritic senile plaque accumulation. The right panel represents Huntington's disease (HD). SIRT1 deacetylates CREB-regulated transcription coactivator 1 (TORC1), which allows TORC1 to activate cAMP response element-binding protein (CREB). CREB then transcriptionally upregulates brain-derived neurotrophic factor (BDNF). The increase in BDNF promotes neurotrophic and neuroprotective mechanisms against HD pathology.

Recent evidence has linked SIRT1 activity with the interference of the factors and-or processes that produce NSP's and NFT's (Figure 7). CR in mice was shown to reduce the expression of β-secretase in part due to the activation of SIRT1 (104). This effect was through the AMPK-SIRT1-PGC-1α pathway in which the transcription factor PGC-1α became upregulated and reciprocally downregulated β-secretase. PGC-1α required SIRT1 deacetylase activity for its transcriptional repression of β-secretase. Thus, CR induced activation of SIRT1 promotes AD neuroprotection through changes in transcription factor activity. As stated before p-tau can contribute to AD. The acetylation of p-tau (acetylated-tau, ac-tau) prevents its degradation and promotes pathological accumulation (105). The overexpression of SIRT1 in HEK293T cells expressing human tau showed a reduction of ac-tau, while SIRT1 deletion results in hyperacetylation. Furthermore, a GST pull-down assay showed a direct interaction of SIRT1 and tau (105). SIRT1 has the capacity to deacetylate ac-tau, which in turn, allows for the degradation of tau and p-tau, potentially reducing the formation of NFT's in AD pathology.

In an oxidative stress model of neuroblastoma SK-N-BE cells, the SIRT1 activator resveratrol was administered to determine its effect on neurodegenerative oxidative stress and protein aggregation (106). Resveratrol treatment prevented toxicity from hydrogen peroxide-induced oxidative stress and prevented Aβ aggregation (106). When applying sirtinol, a non-specific SIRT1 inhibitor, the protection afforded by resveratrol against oxidative stress was lost but not the prevention of Aβ accumulation (106). This evidence indicates that oxidative stress that accompanies AD can be protected against through SIRT1 activation, however, resveratrol protection of Aβ accumulation is SIRT1-independent. SIRT1 overexpression in a transgenic mouse model of AD was investigated by Corpas et al. (107). They studied the CA1 region of the hippocampus to determine if SIRT1 is protective against memory loss and cognitive decline in AD. In the transgenic AD mouse, 6 months of SIRT1 overexpression preserved learning and memory (107). SIRT1 overexpression heavily reduced the presence of Aβ and p-tau in the AD model while increasing the expression of neurotrophic factors, such as brain-derived neurotrophic factor (BDNF) (107). Interestingly, in wild-type mice, SIRT1 overexpression enhanced cognitive function (107). Overexpression of SIRT1 provided protection against pathological protein aggregation and cognitive decline in an AD model while improving cognitive function in the wild-type control (107). SIRT1 is a robust candidate for AD therapies as it has been shown to prevent the accumulation of NSP's and NFT's, reduce AD-related oxidative stress, and protect against the cognitive deficits that result from AD pathology. Further investigation into SIRT1's role in AD protection may provide endogenous targets for treating, and potentially preventing, the disease.

### Parkinson's disease and the therapeutic potential of SIRT1

PD is a neurodegenerative disease that causes the early and large-scale death of dopaminergic neurons (DA) in the substantia nigra pars compacta (SNpc) (108). The loss of these dopaminergic neurons results in motor deficits and other quality of life diminishing symptoms (109). PD pathology is not exclusive to dopaminergic neurons or the SNpc; thus, making PD therapies difficult to design. DA neuronal death in PD typically results from the aggregation of the protein α-synuclein which forms inclusions called Lewy bodies and Lewy neurites (108). The inclusions of α-synuclein are formed in some familial cases of PD due to mutations in the *SNCA* gene which produces the protein in a misfolded state (110). Another prominent aspect of PD is inflammation and reactive gliosis, both of which may have the capacity to be harmful and protective (111). Overall, the current therapies and understanding of pathology for PD are lacking, making PD a pressing focus of future investigation.

Once again, SIRT1 may play a protective role in neurodegenerative disorders, PD included. There is evidence that suggests there may be genetic correlations between SIRT1 and PD, SIRT1-activated anti-PD signaling, and SIRT1-dependent neuroprotection in various models. Extracellular α-synuclein accumulation leads to mitochondrial dysfunction and a reduction of SIRT1 expression (112). The downregulation of SIRT1 facilitated pathological mechanisms, such as apoptotic cell death. In a genetic study with PD patients and healthy controls, the sequence of the SIRT1 promoter and associated regulatory regions were analyzed to determine if there is a mutational connection between the factor and the disease. Three heterozygous sequence variants within the SIRT1 promoter were identified in PD patients, but not controls (113). These variants may alter the transcription of the SIRT1 gene and could potentially link SIRT1-associated mutations to PD risk. These lines of evidence suggest that the loss or mutation of SIRT1 facilitates PD pathology which highlights SIRT1 as a protective target.

There is evidence that shows that cellular signaling resultant from SIRT1 activation, or pathways that include SIRT1, are involved in the reduction of α-synuclein and promotion of DA neuron survival (Figure 7). The application of an activator for the PPARγ, called GW1929, to an *in vitro* human DA neuronal culture conferred resilience when the cultures were subjected to oxidative stress (114). This resilience was attributed to antioxidant signaling and PGC-1α stimulation. GW1929 treatment increased SIRT1 expression and protein levels. GW1929 also resulted in phosphorylated cyclic-AMP response element binding protein 1 (CREB), a pro-survival transcription factor, which then activated SIRT1 (114). Ultimately, upregulation and activation of SIRT1 activated PGC-1α to confer DA neuron protection against oxidative stress (114). In this way, SIRT1 is indirectly upregulated and activated by PPARγ activation in DA neurons suggesting a key role for SIRT1 in DA neuron vitality. In PD, PGC-1α activity may be altered resulting in downregulation of its target genes (115). A study looked at how resveratrol treatment would affect PGC-1α and metabolic homeostasis in primary fibroblasts from early-onset PD. The treatment of resveratrol helped to regulate metabolic homeostasis through AMPK-SIRT1-PGC-1α signaling. An increase in PGC-1α transcription and improvement in mitochondrial function was observed (115). Again, activated SIRT1, in this study through resveratrol treatment, conferred protection against a PD model by activating PGC-1α. In a mouse model of PD induced by 1-methyl-4-phenyl-1,2,3,6-tetrahydropyridine (MPTP), the transgenic overexpression of PGC-1α conferred DA neuronal protection against oxidative stress. Resveratrol treatment recapitulated the protective effects of PGC-1α overexpression in the mouse PD model (116). In a PC12 PD model, the treatment of EGCG, a polyphenol, protected against toxicity through an upregulation of PGC-1α, via SIRT1 activity (117). It is clear that SIRT1 signaling can result in the activation of PGC-1α which is protective against oxidative stress and PD pathology. The evidence supports SIRT1 as a target for future therapeutic approaches in PD treatment due to its induction of neuroprotective cell signaling. Further investigation will provide a greater depth of understanding for PD pathology.

Inflammation within the CNS can exacerbate or potentially initiate PD pathology (118). The cell signaling that occurs from increased inflammation and reactive nitrogen species enhances the dysfunction of neurons and promotes cell death (119). A study looked at how inducible nitric oxide synthase (iNOS), which produces NO that can modify proteins through S-nitrosylation, effects inflammatory signaling in neurodegenerative diseases. S-nitrosylation of SIRT1 inhibits its deacetylase activity. In a rodent model of PD with systemic inflammation, S-nitrosylation of SIRT1 correlated with an increase in p53 and NF-κB acetylation, thereby increasing their activity and promoting further inflammation (119). In SH-SY5Y cells, SIRT1 was shown to directly deacetylate histone residue H3K9 of the p53 promoter, eventually resulting in reduced expression and protecting against apoptosis (120). Additionally, resveratrol-activated SIRT1 regulated p53 and protected against dopaminergic neurodegeneration induced by rotenone, a complex I inhibitor (120). As an inflammatory regulator in PD and potentially other neurodegenerative diseases, SIRT1's deacetylase activity protects against pro-apoptotic inflammatory signaling.

The removal of α-synuclein in a healthy DA neuron entails the use of a few cellular clearance mechanisms, most of which involve some type of autophagy (121). One of these mechanisms utilizes a recruitment protein called LC3, which helps to drive degradation of misfolded α-synuclein that is present in the LC3 bound autophagosome (122). In an MPTP-mouse model, resveratrol or EX-527 were administered to study their effects on motor impairments and autophagic clearance of α-synuclein (122). Resveratrol treatment attenuated MPTP effects on motor deficits and autophagic impairment while EX-527 exacerbated them (122). Furthermore, the beneficial effects of resveratrol treatment were shown to be SIRT1 dependent. SIRT1 was shown to deacetylate nuclear LC3 allowing for its translocation to the cytosol from the nucleus and initiate autophagic clearance (122). Thus, SIRT1 deacetylase activity mediates clearance of α-synuclein through LC3 mediated autophagy to protect against PD pathology. In addition, activation of the AMPK-SIRT1-autophagy pathway was shown to increase LC3-II and enhance α-synuclein clearance after resveratrol treatment (123). Increased clearance of α-synuclein is yet another mechanism by which SIRT1 confers protection against PD pathology.

Considering the multitude of evidence supporting SIRT1's neuroprotective potential against PD and the dynamic range of mechanisms in which that protection is enacted, SIRT1 appears to be an optimal target for the therapeutic treatment of PD. However, recent attempts to enhance SIRT1 expression or activity directly, not through activators, has not shown the same robust results. A study utilized a CNS SIRT1 overexpression mouse model to study the MPTP model of PD. As compared to controls the SIRT1 overexpression mouse did not confer protection against acute toxicity of MPTP in nigrostriatal DA neurons (124). Additionally, in a study that looked at the modulation of SIRT1 expression in multiple human neurodegenerative diseases, there was no significant change found in the SIRT1 expression of patient samples of PD and Lewy bodies dementia (125). This contradictory evidence suggests SIRT1 must operate in a network of cellular signaling and deacetylase activity to confer is neuroprotection against PD pathology.

### Huntington's disease and the therapeutic potential of SIRT1

Another neurodegenerative disease in which SIRT1 has been investigated is HD. HD is a genetically autosomal dominant disease in which the HD gene produces a mutant version of the protein. Extended CAG-repeats in the HD gene results in the translated protein acquiring a pathological conformation, affecting its solubility and promoting aggregations (126). Aggregations of the pathological Huntington's protein commonly occur in the axons of neurons, predominantly within the striatum. These axonal aggregations are considered to block anterograde and retrograde axonal transport in affected cells. Post-mortem tissue of HD patients showed cytological features of ballooned cells and shrunken cells within the affected brain regions (127). The blockage of axonal transport is suggested to result in pathological localization of mitochondria and mitochondrial dysfunction. The combination the cytological aberrations and mitochondrial dysfunction are implicated as leading reasons for neuronal death in HD pathology (128).

Like other neurodegenerative diseases, the role of SIRT1 has been investigated in the context of HD pathology. In the R6/2 mouse model of HD, the levels of metabolic and cell cycle regulators were assessed. SIRT1 mRNA and protein were increased in this model but this increase did not correlate to increased activity as shown by no significant change in p53 acetylation (129). This change in SIRT1 expression suggests that SIRT1 levels are altered as a result of HD pathology. In the same model of HD, treatment with β-Lapachone, a natural compound found in the Lapacho tree's bark, was shown to increase the expression of SIRT1 (130). Increased SIRT1 resulted in PGC-1α deacetylation and CREB phosphorylation, which correlated with reduced reactive oxygen species and improvement of rota-rod performance. β-Lapachone thus showed therapeutic potential for HD, enacted through SIRT1 activation. There may be specific contexts in which increased SIRT1 is therapeutic rather than a feature of HD pathology and this is likely related to increased SIRT1 activity.

Many studies have intentionally augmented SIRT1 in the contexts of HD to elucidate whether it is part of the pathology or potentially therapeutic. In an HD mouse model, SIRT1 was overexpressed, improving motor functions and pathological metabolic functioning (131). SIRT1 overexpression was shown to alleviate the HD associated reduction in BDNF concentrations (Figure 7). BDNF signaling via its TrkB receptor was shown to be rescued as well. Interestingly, this study also suggests that mutant HD protein inhibits deacetylase activity of SIRT1, as shown by the hyperacetylation of SIRT1-specific targets in the presence of HD mutant protein (131). Another study looked at the effects of SIRT1 absence in HD pathology by using a brain-specific KO of SIRT1 in a mouse model of HD (132). The loss of SIRT1 exacerbated pathological features of HD. These mice had acceleration of motor deficits and increased mutant HD protein aggregation compared to the HD mice with SIRT1 (132). This study also investigated SIRT1 overexpression, which afforded neuroprotection against HD. SIRT1 neuroprotection was dependent on CREB-regulated transcription coactivator 1 (TORC1), which is deacetylated by SIRT1. This interaction increases BDNF and in the presence of the mutant HD protein, the SIRT1-TORC1 interaction is inhibited, repressing BDNF (132).

The role of SIRT1 in HD certainly warrants further investigation. Though there are pathological increases in SIRT1 in neurons suffering from HD pathology, this may be a compensatory mechanism due to the inhibition of SIRT1 by the mutant HD protein. In studies overexpressing SIRT1, HD pathology has been ameliorated and this protection is dependent on SIRT1's activity. Taken together, these lines of evidence suggest that SIRT1 is inhibited in HD pathology and there may be an increase in its expression for compensatory reasons. Furthermore, the loss of SIRT1 deacetylation activity may contribute to HD pathology and restoration of SIRT1 activity likely possess therapeutic potential against the disease.

## Conclusions

In summary, any dysregulation of brain SIRT1 activity can have devastating consequences in terms of metabolism, circadian synchronization, and neurological function. Given that SIRT1 is highly specialized distributed in the hypothalamic nuclei, it is no surprise that brain SIRT1 is a major contributor to the systemic network of metabolic homeostasis. It should be noted that, nowadays, accumulated evidence supports a reciprocal relationship between brain and peripheral tissues in metabolic benefits, circadian oscillations and neurological functions (133, 134). Although we only discussed SIRT1 in the brain, SIRT1 in various peripheral organs also mediates metabolism and circadian rhythms through sensing environmental cues and feeding back into the homeostatic network (13, 133). Therefore, pharmacological agents that target SIRT1 and its relevant signal pathway in one system could potentially provide pleiotropic benefits.

Although most studies discussed above have used resveratrol as the SIRT1 activator, limitations remain for the resveratrol-induced SIRT1 activation. One study reported the activation of AMPK by high-dose resveratrol, suggesting the indirect effects of SIRT1 through AMPK pathway as well as the off-target effects of resveratrol (135). As a polyphenol activator, resveratrol is poorly water-soluble. Thus, the bioavailability of resveratrol also needs to be taken into consideration, especially when applied in clinical trials. To improve the bioavailability, targeted delivery of resveratrol, such as nanoparticles has been developed (136, 137).

In addition to resveratrol, another promising target to activate SIRT1 is the NAD^+^ pathway. As mentioned before, NAD^+^ is the rate-limiting co-substrate for SIRT1. Thus, increased NAD^+^ levels is presumably to activate SIRT1. So far, to supplement NAD^+^ precursor and boost NAD biosynthesis has been the main approaches to alter NAD^+^ levels. Experimental studies have reported beneficial effects of NAD^+^ precursor supplementation (138–140). Current information from clinical studies is still lacking. One recent study reported promising result that chronic nicotinamide riboside, a NAD^+^ precursor, effectively increased NAD^+^ levels in elders (141). Again, it is difficult to tell whether the beneficial phenotypes are produced by NAD^+^ or NAD^+^-induced SIRT1 activation. Therefore, pharmacological agents that are ligand-specific and tissue-specific are warranted to further clarify the functions of SIRT1 in biological and pathological events.

## Author contributions

JX, CJ, NK, IE, and MP-P cooperated to write the article and revised the content. MP-P and JX outlined the manuscript. MP-P supervised the work.

### Conflict of interest statement

The authors declare that the research was conducted in the absence of any commercial or financial relationships that could be construed as a potential conflict of interest.
